# Genetic Diversity of Grasspea and Its Relative Species Revealed by SSR Markers

**DOI:** 10.1371/journal.pone.0118542

**Published:** 2015-03-20

**Authors:** Fang Wang, Tao Yang, Marina Burlyaeva, Ling Li, Junye Jiang, Li Fang, Robert Redden, Xuxiao Zong

**Affiliations:** 1 The National Key Facility for Crop Gene Resources and Genetic Improvement/Institute of Crop Science, Chinese Academy of Agricultural Sciences, Beijing 100081, China; 2 Department of Leguminous Crops Genetic Resources, N. I. Vavilov Research Institute of Plant Industry, St. Petersburg 190000, Russia; 3 Institute of Cash Crops, Liaoning Academy of Agricultural Sciences, Liaoyang 111000, China; 4 Australian Temperate Field Crops Collection, Grains Innovation Park, The Department of Primary Industries, Horsham, Victoria 3401, Australia

## Abstract

The study of genetic diversity between *Lathyrus sativus* L. and its relative species may yield fundamental insights into evolutionary history and provide options to meet the challenge of climate changes. 30 SSR loci were employed to assess the genetic diversity and population structure of 283 individuals from wild and domesticated populations from Africa, Europe, Asia and ICARDA. The allele number per loci ranged from 3 to 14. The average gene diversity index and average polymorphism information content (PIC) was 0.5340 and 0.4817, respectively. A model based population structure analysis divided the germplasm resources into three subgroups: the relative species, the grasspea from Asia, and the grasspea from Europe and Africa. The UPGMA dendrogram and PCA cluster also demonstrated that Asian group was convincingly separated from the other group. The AMOVA result showed that the cultivated species was quite distinct from its relative species, however a low level of differentiation was revealed among their geographic origins. In all, these results provided a molecular basis for understanding genetic diversity of *L*. *sativus* and its relatives.

## Introduction

The genus *Lathyrus* L. includes as many as 187 species [1,2]. These are distributed throughout temperate regions of the Northern Hemisphere and extend into tropical East Africa and South America. However, the main centers of diversity include the Mediterranean and Irano-Turanian regions [3]. Grasspea (*Lathyrus sativus* L.) is the only species widely cultivated as a food crop in the genus *Lathyrus*, whereas other species (*Lathyrus cicera* L. and *Lathyrus ochrus* L.) are cultivated to a lesser extent [4]. Moreover, grasspea has great agronomic potential as a grain and forage legume in the fragile agro-ecosystems, because of its ability to survive under extreme climatic conditions such as drought, flood and salinity [5].

There have been recent studies of genetic diversity in *Lathyrus sativus*. PCR-based molecular markers utilized so far in *L*. *sativus* and its relative species include random amplification of polymorphic DNA (RAPD) [6,7], restriction fragment length polymorphism (RFLP) [8] which was indicated the highly similarity between *L*. *sylvestris* L. and *L*. *latifolius* L., amplified fragment length polymorphism (AFLP) [9] clarified that 20 central Italy grasspea accessions were divided into the Household populations and the Commercial populations which was useful for the grasspea bereeding in central Italy, and inter-simple sequence repeat (ISSR) was used for exploring the genetic diversity among *L*. *sativus*, *L*. *cicera*, and *L*. *ochrus* [10].

Up to now, there was little study of genetic diversity in *Lathyrus sativus* using simple repeat sequence (SSR) markers [11–13] (Table 1). Lioi et al. searched for EST sequences of *L*. *sativus* with the European Molecular Biology Laboratory (EMBL) nucleotide sequence database. Amplification was successful only in 10 out of 20 of the SSR primers, with only 6 of these exhibiting size polymorphism and subsequently used in genetic diversity analysis for 13 Italian landraces [11]. Shiferaw et al. used 11 EST-SSRs developed from *L*. *sativus*. EST-SSRs derived from *Medicago truncatula* L. to investigate the genetic diversity among 20 grasspea accessions from Ethiopia [12].

**Table 1:** SSR markers used in grasspea researches from literature and this study.

The origin of the primers	Type	Number of primers	Number of polymorphism primers
**Lioi et al. (2011)**	SSR	20	6
**Shiferaw et al. (2012)**	EST-SSR	43	11
**Yang et al. (2014)**	SSR	284	74
**This study**	SSR	120	30

**Fig 1:**
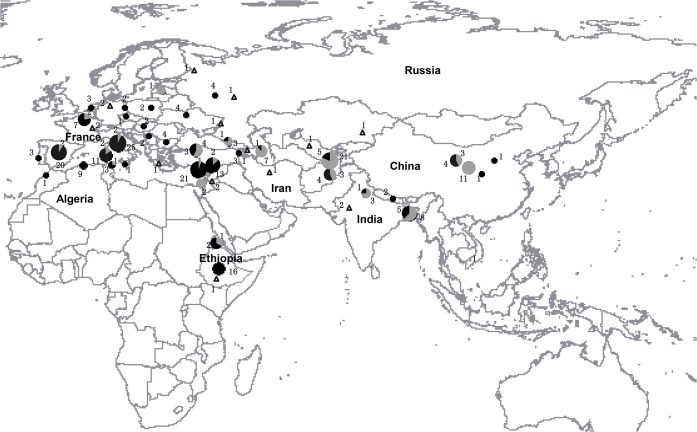
Geographic distribution of *Lathyrus sativus* based on the results of structure analysis and the *Lathyrus sativus* relative species. Gray indicates accessions from Asia, and black means the accessions from Africa/Europe as respective proportion of circles for distribution of number of accessions at each location; the hollow triangle means the distribution of *L*. *sativus* relative species.

Using the 454 FLX Titanium pyrosequencing technique, a large-scale microsatellite approach was developed in *Lathyrus sativus* [13]. Potentially these SSR primers can make a significant contribution to genomics enabled improvement of grasspea. To broaden the genetic variation of cultivated grasspea in the future for China, it is necessary to perform a more comprehensive analysis of genetic diversity and population structure in the national genebank. We used 30 polymorphic genomic-SSR markers developed by Institute of Crop Science, Chinese Academy of Agricultural Sciences, Beijing, China (ICS/CAAS) [13], to study the genetic diversity among 266 accessions from *L*. *sativus* and 17 accessions from its cultivated and wild relatives (Fig. 1).

## Materials and methods

### Plant materials

A total of 266 grasspea accessions (Table 2) and 17 relative accessions (Table 3) were collected and tested in the protected field of experimental farm within CAAS campus (39° 57' 38" N, 116° 19' 27" E). For *Lathyrus sativus*, European germplasm comprised 100 accessions from 14 countries, while Asian germplasm contained 20 accessions from China and 98 non-Chinese accessions. African germplasm included 33 accessions and ICARDA comprised 15 accessions (Fig. 1). The 17 accessions of 9 relative species are from Europe, Asia, and Africa (Fig. 1). Seed supplies direct from collected samples were sourced from ICS/CAAS, as well as from N. I. Vavilov Research Institute of Plant Industry, St. Petersburg, Russia. Maps of the genus *Lathyrus* collection sites were conducted with DIVA-GIS based on latitude and longitude coordinates [14].

**Table 2:** Geographic origin of 266 *Lathyrus sativus* accessions used in this study.

Origin	Country of origin	Number of accessions	Longitude	Latitude
**Africa**	Algeria	9	3.133	36.700
	Eritrea	3	38.550	15.200
	Ethiopia	16	38.990	8.533
	Morocco	1	-6.850	34.033
	Tunisia	4	10.183	36.833
**Europe**	Bulgaria	4	24.933	42.950
	Czech	1	14.250	50.050
	Former Yugoslavia	2	20.467	44.817
	France	9	2.993	48.833
	Germany	2	13.997	52.500
	Holland	3	4.900	52.383
	Hungary	2	19.083	47.483
	Island Sardinia, Italy	13	9.117	39.217
	Island Sicily, Italy	1	14.000	37.000
	Italy	27	12.483	41.900
	Latvia	1	24.060	56.560
	Poland	2	21.000	52.217
	Portugal	3	-9.167	38.700
	Russia	4	37.983	55.750
	Spain	22	-3.750	40.417
	Ukraine	4	30.483	50.467
**Asia**	Afghanistan	7	69.183	34.467
	Armenia	3	44.310	40.110
	Azerbaijan	8	49.990	40.260
	Bangladesh	13	90.240	23.420
	Gansu, China	7	103.823	36.078
	Ningxia, China	11	106.250	36.017
	Shaanxi, China	1	108.944	34.265
	Shanxi, China	1	112.551	37.871
	Georgia	4	44.793	41.710
	India	4	78.200	28.617
	Island Cyprus	22	33.417	35.167
	Nepal	2	85.317	27.700
	Palestine	2	34.467	31.500
	Tajikistan	26	68.470	38.320
	Turkey	7	32.900	39.950
**ICARDA**	Syria	15	37.159	36.217

**Table 3:** Geographic origin of 17 accessions from nine different *Lathyrus sativus* relative species used in this study.

Species	Growth habit	Country of origin	Number of accessions	Longitude	Latitude
*Lathyrus aphaca* L.	Annual	India	1	77.017	28.617
		Krasnodar region, Russia	1	38.983	45.033
*Lathyrus cicera* L.	Annual	Germany	1	13.017	52.500
		Ethiopia	1	37.500	8.533
		Syria	2	36.300	33.500
*Lathyrus clymenum* L.	Annual	Germany	1	13.017	52.500
		Greece	1	23.767	37.967
*Lathyrus hirsutus* L.	Annual	Uzbekistan	1	69.130	41.160
*Lathyrus ochrus* (L.) DC	Annual	India	1	77.017	28.617
	Annual	Iran	1	51.500	35.733
*Lathyrus tingitanus* L.	Annual	France	1	2.033	48.833
*Lathyrus latifolius* L.	Perennial	France	1	2.033	48.833
*Lathyrus pratensis* L.	Perennial	Kazakhstan	1	38.983	45.033
		Vologda region, Russia	1	37.083	55.750
*Lathyrus sylvestris* L.	Perennial	Azerbaijan	1	49.020	40.260
		Leningrad, Russia	1	30.417	59.917

### DNA extraction

Genomic DNA was extracted from pooled ten random young seedlings of each accession using the CTAB method [15,16] with 1% PVP added.

### Polymerase chain reactions (PCR) amplification

Polymerase chain reactions (PCR) were performed in 10 μl reaction volumes containing 5 μl 2 x TaqPCR MasterMix (Hooseen, Beijing, China), 1 μl primer, 1.5 μl of genomic DNA (30 ng) and dd H_2_O 2.5 μl. Microsatellite loci were amplified on a K960 Thermal Cycler (Jingle, Hangzhou, China) with the following cycle: 5 min initial denaturation at 95°C; 35 cycles of 30 s at 95°C, 30 s at the optimized annealing temperature (Table 4), 45 s of elongation at 72°C, and a final extension at 72°C for 10 min. The PCR products were separated on 8% non-denaturing polyacrylamide gel electrophoresed under 280 V and 50 W and visualized by 0.1% silver nitrate staining.

**Table 4:** Characteristics of 30 polymorphic microsatellite loci used in this study (FP = forward primer, RP = reverse primer, Ta = annealing temperature).

Primer	Repeat motif	Primer sequence(5’-3’)	Real product size(bp)	T_a_ /°C
G5	(AAC)10	FP-CACAACCAGTTGCATCAGTG RP-TGGCTCACATGATGGTTTGT	200–220	54
G9	(AAC)6	FP-CAACCAGAGCAACCACAAGA RP-GGTTGCAAGAGGTTGCAGAT	200–260	53
G17	(AAT)5	FP-CAGGTCCGGCTTATCTCTCA RP-TTGGTTTCAACCCACTCCTC	195–240	52
G26	(AC)16	FP-CCACCAAATTTCCCTTTTTG RP-GGTACGAGAGGTTGACTTTTGTTT	170–200	52
G49	(AC)7	FP-ACGCACACACGGAAGAAAG RP-GTGTGCGCATGTGTGTATGA	180–195	58
G67	(AC)9	FP-CACCCTCTTCACTGCCTAGC RP-TTGGGGGTTGTAGAAGGAAC	135–150	52
G68	(AC)9	FP-GCACACAAGGGCACACTG RP-TGCGTCGTGTGTATGTGTTG	180–220	52
G116	(CA)6(CACACG)5	FP-CACACAGGACAGCACTCACA RP-GTCGTCGGTGTGTCGTAGTC	140–175	56
G131	(CA)7aacacgttcg(CA)8	FP-GCGCTCACACCAACATAAAG RP-TGTATGCGTGCGTATGTCTG	150–160	54
G157	(CAA)6	FP-ACATCCAATCCCCACCATAA RP-AATGCATGGTTGTTGCTTGA	210–220	60
G163	(CAC)6	FP-CAGTAGCATCAACAACGACTCC RP-GTTGTGCCATGTGTTGTGTG	140–160	52
G185	(GT)19	FP-TGCGTGTGTCGCTCTATCAT RP-TACTGCGACAACCGAACGTA	120–130	52
G200	(GT)7	FP-GGATGGTGTGCTGTGTGTGT RP-AACACCAACTACCGGCAACT	140–150	52
G206	(GT)8	FP-AAACTGGCCCTGCATTTTC RP-GGTCATGGCAATTTGAGACA	180–195	52
G213	(GT)9c(GT)7	FP-TTTGTGTCACAGCCCTGTTT RP-CATGTTGGCTGCAAGTTTGT	170–180	52
G245	(TG)6	FP-CGTTGGTTGTTAGTCGGTCA RP-GAACGAAACAACGACGACAA	220–240	52
G285	(TTG)6	FP-TTTGTGCGGTTGATGTTGTT RP-CTACGTCAGCCCGTCATACC	195–220	52
G15624	(AAC)11	FP-GCAACAACAAATGCAACATC RP-TGTTGTTACTGCTGCTGCTCT	150–170	52
G15709	(CAT)5	FP-GACCTCGAGGGACATTAGCA RP-CCAAAGAAAGAGAAAGGACACAA	130–150	52
G15771	(TCG)5	FP-AGTGCCTGATGGGAGTCAGT RP-CCGACGACGACGACTACTAA	200–230	56
G17243	(GTC)5	FP-GCGTGTGTCGTCGTGTAGTT RP-GCCGTACGACACCAAGTACC	140–180	52
G17922	(CCA)5	FP-CACCACCATAACCACCTCCT RP-ATGCGATTGAAGGGATGAAC	180–220	52
G18078	(TGT)8	FP-TTCAGATGCAGGTGGTTCAG RP-AACGGTGCGACTCTTGCTAT	140–150	52
G18109	(CGA)5	FP-GACAGACACACGGCAAACAC RP-ACGTCGTCGTGTCGTTGTT	170–200	52
G18200	(AAC)5	FP-CAACACAACACAACAACACGAT RP-CAGTCACGTCCCTCAGTGC	90–100	52
G18308	(AAC)5	FP-CAATATACAAGCAACCACACCAC RP-TGTTGCGTCTAATTGTTGTGTTC	185–195	52
G18549	(GTT)5	FP-TGAGGGTGTTTGAACGTGAG RP-CACCACAACAACAACAACCAC	140–170	52
G19207	(AAG)5	FP-ATCGTAAACCGTGAGGGTCA RP-AAGCTTGTGGTGGCTACTGC	200–210	54
G19337	(ACA)5	FP-CGACAACACATACAGCAACAC RP-TGTTGTTCGTTGTTGTTAGTTAGTT	220–240	52
G19347	(GAA)5	FP-CCTCTCTCCGCAATCTTGTC RP-CGTTCATCATCCATATCATCCT	110–120	52

### Data analysis

The genetic diversity parameters and polymorphism information content (PIC) of each primer pair were calculated by Powermarker v3.25 [17] using the following formulas: Gene diversity: $\text{D}=(1-\sum_{u=1}^{k}p_{1u}^{2})/\left(1-\left(1+\text{f}\right)/\text{n}\right)$; PIC = ∑(1—pi^2^)/n, where pi is the frequency of the ith allele, n is the total number of genotypes [18]. POPGENE version 1.32 [19] was used to calculate Nei's genetic distance [20]. The program STRUCTURE V2.3.3 [21,22] was used to examine population structure and differentiation. The simulations were run with a burn-in of 100,000 iterations and from K = 1 through 10. Runs for each K were replicated 160 times and the true K was determined according to the method described by Evanno et al. [23]. The number of subgroups (K) was identified based on maximum likelihood and delta K (ΔK) values. The cluster analysis of different geographical groups was carried out using unweighted pair-group method with arithmetic average (UPGMA), and the dendrogram was drawn by MEGA 5.02 [24]. Analysis of molecular variance (AMOVA) was used to assess the variance among and within populations from different geographical origin with GenAlEx 6.41 software [25]. Principal component analysis (PCA) was applied to show the distribution of individual accessions in scatter diagram and two-dimension PCA graph was drawn using the NTSYSpc 2.2 statistical package [26].

## Results

### SSRs polymorphic testing

120 SSR markers were randomly selected to validate polymorphism at first. 25% of them were polymorphic (Table 4). 30 SSR makers amplified 258 polymorphic bands with an average of 8.6, ranged from 3 to 14 per primer pair (Table 5). Gene diversity was from 0.0708 to 0.8505, and the average was 0.5340. Meanwhile, polymorphism information content (PIC) of each primer pair ranged from 0.0688 to 0.8338 with an average of 0.4817. These results demonstrated polymorphic SSR markers which we used were good enough for further genetic diversity analysis.

**Table 5:** Results of primer screening through 283 diversified accessions in genus *Lathyrus*.

Marker	Allele No.	Gene Diversity	PIC
G5	10	0.6761	0.6253
G9	7	0.6036	0.5284
G17	13	0.4777	0.4245
G26	11	0.8242	0.8017
G49	7	0.4427	0.4094
G67	13	0.8505	0.8338
G68	10	0.4838	0.4107
G116	7	0.3710	0.3157
G131	8	0.4561	0.4374
G157	6	0.6484	0.5849
G163	9	0.5591	0.4940
G185	3	0.0708	0.0688
G200	8	0.5680	0.4763
G206	7	0.4431	0.3652
G213	9	0.4321	0.4032
G245	9	0.5621	0.4881
G285	9	0.2483	0.2351
G15624	10	0.3125	0.2944
G15709	9	0.2789	0.2702
G15771	8	0.5793	0.5109
G17243	8	0.5529	0.4578
G17922	11	0.6885	0.6388
G18078	8	0.6818	0.6269
G18109	14	0.7624	0.7292
G18200	9	0.4691	0.4178
G18308	6	0.5845	0.5223
G18549	7	0.5872	0.5321
G19207	8	0.6563	0.5880
G19337	10	0.6397	0.5693
G19347	4	0.5081	0.3896
Mean	8.6	0.5340	0.4817

### Genetic diversity and classification analysis among populations of *Lathyrus sativus* and its relative species

**Fig 2:**
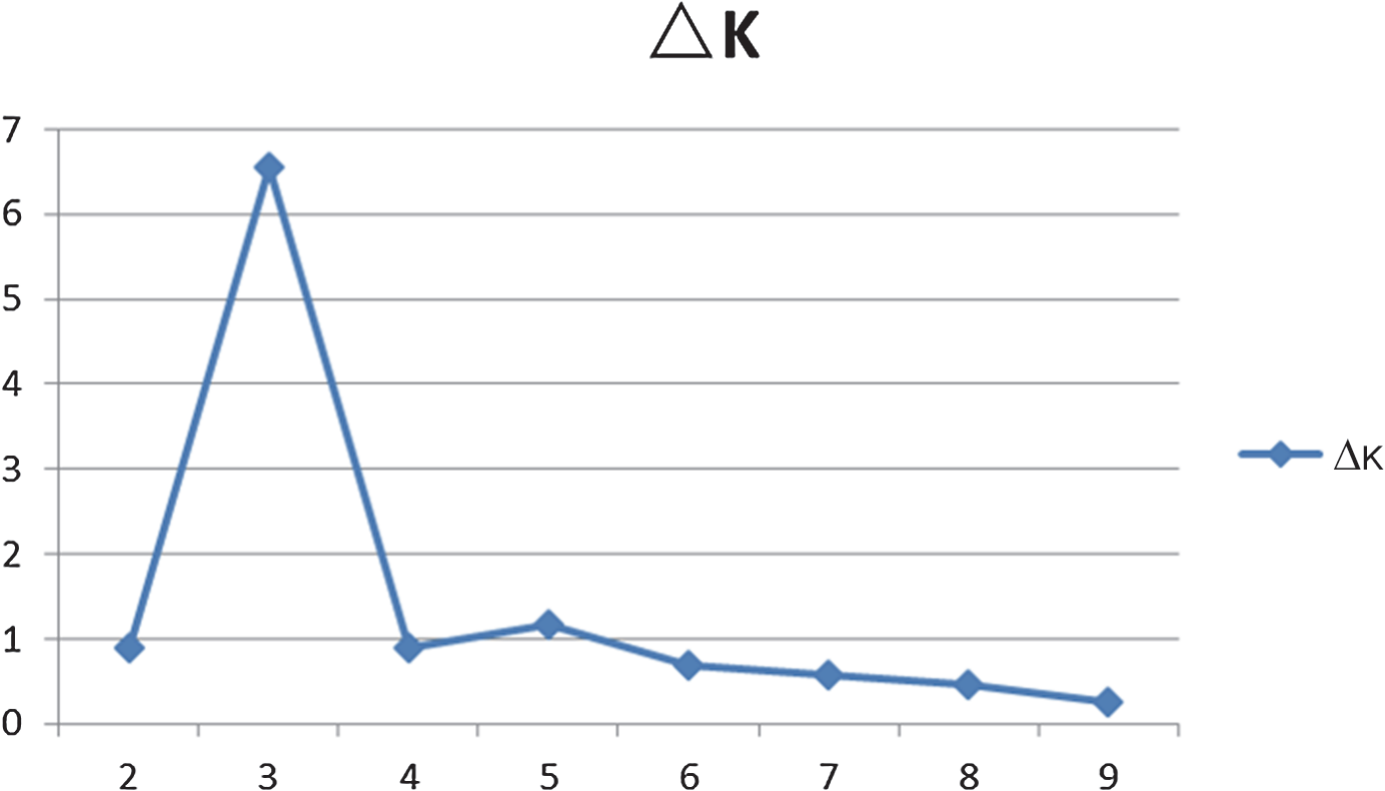
ΔK was used to determine the most appropriate K value for population structure in the *Lathyrus* genus.

**Fig 3:**
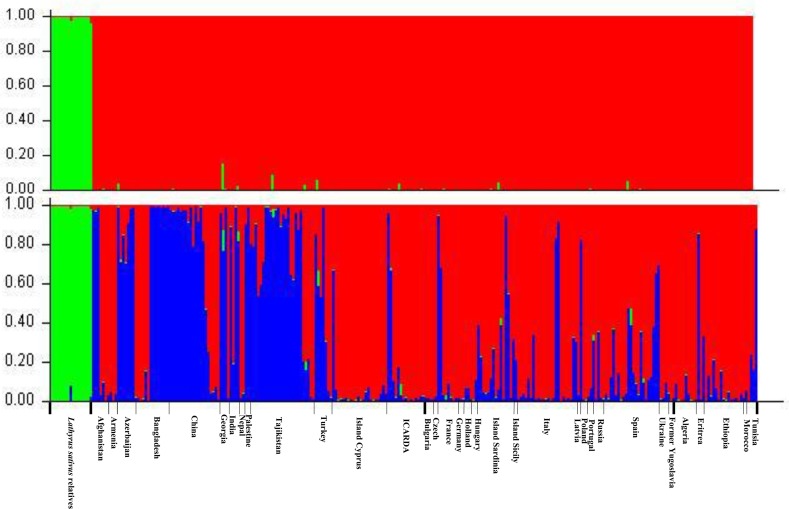
Population structure of K = 3 inferred by Bayesian clustering approaches based on 30 microsatellite markers showing relatives of *L*. *sativus* and separation of *L*. *sativus* into Asian and African/European subgroups.

The population structure of *Lathyrus sativus* and its relatives was inferred by using STRUCTURE V2.3.3 based on 30 SSR markers. At K = 2, all the germplasm were divided into *L*. *sativus* and its relatives. But, according to the method described by Evanno et al. [23], three populations should be identified theoretically based on delta K (ΔK) values (Fig. 2), therefore the genetic structure of 283 accessions can be described with greatest probability and no gain in discrimination. At K = 3, the related accessions were in one subgroup and the *L*. *sativus* also divided into 2 subgroups (Fig. 3). One subgroup contained 79 accessions mainly from Asia. The other subgroup contained 187 accessions and most of them came from European and African countries.

### Genetic relationships analysis

**Fig 4:**
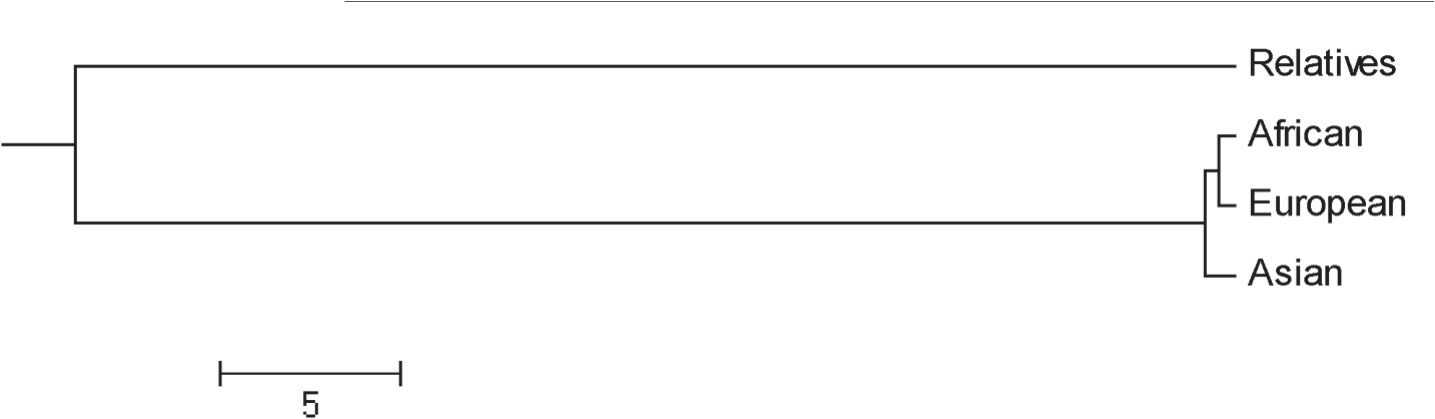
UPGMA dendrogram of Nei’s (1978) Genetic Distance among all *Lathyrus* accessions used in this study.

The *Lathyrus sativus* relatives as a group were marginally more similar to the Asian than to the African and European sources of *L*. *sativus*, whereas the African and European sources of *L*. *sativus* were more closely related than either to the Asian source (Table 6, Fig. 4). All *Lathyrus* accessions were clustered according to Nei’s genetic distance [20] (Fig. 4). The largest genetic distance (0.6360) was between *Lathyrus sativus* relatives and European grasspea, and the smallest genetic distance (0.0038) was between African and European grasspea. Based on the origin of *L*. *sativus* accessions, the genetic distance between Africa and Asia (0.0141) was larger than it between Europe and Asia (0.0118). These result matched with structure analysis above.

**Table 6:** Pairwise estimated of genetic identity and genetic distance based on 30 SSR markers among relatives (17 accessions), African (33 accessions), Asian (133 accessions) and European (100 accessions) of *Lathyrus sativus*.

Pop ID	Relatives	African	Asian
African	0.6234		
Asian	0.581	0.0141	
European	0.636	0.0038	0.0118

Note: Nei's (1978) genetic distance

There were 17 accessions from nine different relative *Lathyrus sativus* species used in this study. Nei’s genetic distance of 0.7247 between *L*. *sativus* and *L*. *cicera* was the smallest in our study among *L*. *sativus* and its nine relative species (Table 7). This result matched morphological [27] and cytogenetical [28] researches which suggest that *L*. *cicera* is the most probable progenitor of *L*. *sativus*. Among *L*. *sativus* relative species, the relationship between *L*. *latifolius* and *L*. *sylvestris* was the closest (Table 7). Meanwhile, the closer phylogenetic relationship between *L*. *latifolius* and *L*. *sylvestris* revealed in our research was also detected by Ceccarelli et al. [29] using satellite DNA and Asmussen and Liston [30] using chloroplast DNA study.

**Table 7:** Pairwise estimated of Nei’s genetic distance based on 30 SSR markers among *Lathyrus sativus* and 17 relative species accessions.

Pop ID	*L*. *sativus*	*L*. *cicera*	*L*. *tingitanus*	*L*. *aphaca*	*L*. *hirsutus*	*L*. *clymenum*	*L*. *ochrus*	*L*. *pratensis*	*L*. *sylvestris*
*L. cicera*	0.7247								
*L. tingitanus*	1.1105	1.0943							
*L. aphaca*	1.2139	1.069	0.9723						
*L. hirsutus*	1.0444	1.0949	1.0797	0.7182					
*L. clymenum*	1.1946	1.1897	1.2229	1.0269	1.0773				
*L. ochrus*	1.1302	1.3884	1.3589	1.55	1.0191	0.8923			
*L. pratensis*	1.1115	1.2094	1.3753	1.2696	1.2563	1.2526	0.9698		
*L. sylvestris*	1.0443	1.003	1.3242	1.2186	0.954	1.137	0.9454	1.0731	
*L. latifolius*	1.4407	1.2208	1.2298	1.4296	0.8973	0.9473	1.1538	1.0187	0.6698

**Fig 5:**
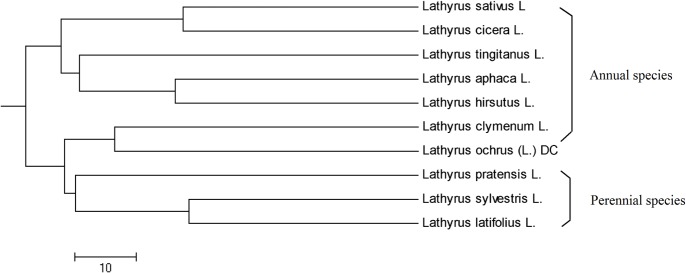
UPGMA dendrogram of Nei’s (1978) Genetic Distance among *Lathyrus sativus* and its relatives.

Clustering analysis based on Nei’s genetic distance divided all the 10 species under genus *Lathyrus* accessions into two major groups (Fig. 5). One group included *L*. *sativus*, *L*. *cicera* L., *L*. *tingitanus* L., *L*. *aphaca* L., and *L*. *hirsutus* L., which were all annual species. The second group comprised *Lathyrus clymenum* L., *L*. *ochrus* (L.) DC, *L*. *pratensis* L., *L*. *sylvestris* L. and *L*. *latifolius* L. In general, *L*. *clymenum* and *L*. *ochrus* were annual species, however, *L*. *pratensis*, *L*. *sylvestris*, and *L*. *latifolius* were perennial species.

### Classification and PCA analysis of all the accessions used in this study

**Fig 6:**
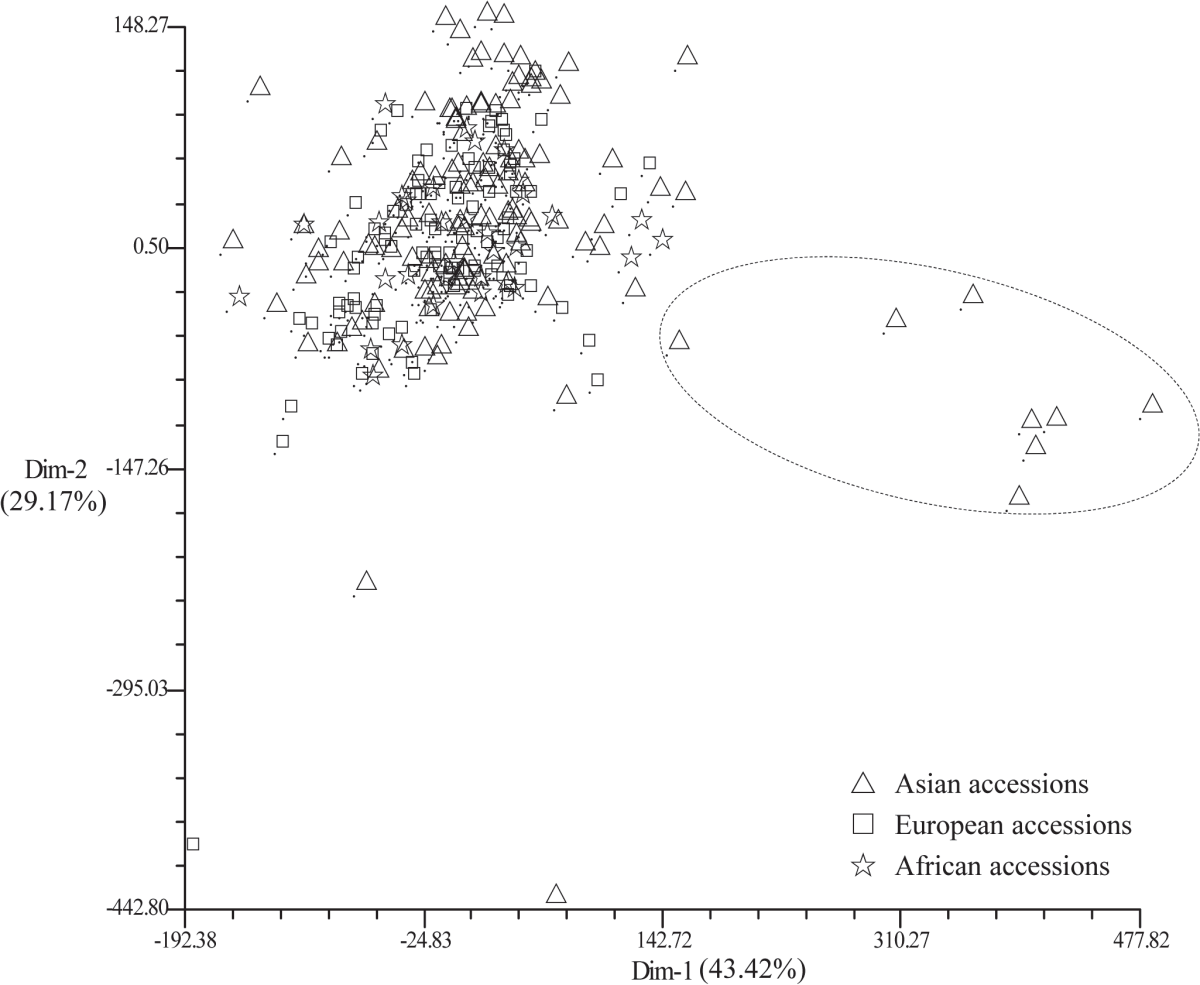
Two-dimension principal component analysis (PCA) of *Lathyrus sativus*. Asia accessions (hollow triangle), and European accessions (open square) and African accessions (open pentagram) are based on the geographical origin.

The genetic relationship of individual accessions was analyzed using principal component analysis (PCA); all the cultivated accessions were labeled according to their geographical origin. Within cultivated species, accessions from Asia were somewhat associated with their geographical origin and were different from other accessions (Fig. 6), especially, eight accessions from Bangladesh were quite apart from African and European accessions. The first two principal components explained 43.42% and 29.17% of the molecular variance, respectively.

### Analysis of molecular variance

First of all, we evaluated genetic differentiation between *Lathyrus sativus* and its relatives by analysis of molecular variance (AMOVA). The results showed that the cultivated species was significantly distinct from its relatives at P-value of 0.0001 (Table 8). Among population variance explained 40% and within population explained 60% of genetic diversity. Secondly, significant genetic differentiation among the three population structure classified subgroups was detected by AMOVA at P-value of 0.0001 (Table 9). The results of AMOVA also indicated that the majority of the genetic variation among all the 283 accessions was due to within population variation (84%). Finally, we evaluated the genetic differentiation among accessions of grasspea (Table 10). The results show a low level of differentiation (3%) among Asia, Europe, and Africa.

**Table 8:** Analysis of genetic differentiation between *Lathyrus sativus* and its relatives by AMOVA.

Source of variation	df	SS	MS	Est. Var.	%	P-value
Species						
Among Pops	1	374.479	374.479	11.186	40	0.0001
Within Pops	281	4776.719	16.999	16.999	60	0.0001

Note: df means degrees of freedom, SS means sum of squares deviations, MS means squares deviations, Est. Var means estimates of variance components, % means percentage of total variance contributed by each component, P-value means probability value.

**Table 9:** Analysis of genetic differentiation among all the accessions based on structure by AMOVA.

Source of variation	df	SS	MS	Est. Var.	%	P-value
Model-based population						
Among Pops	2	478.546	239.273	3.265	16	0.0001
Within Pops	280	4672.652	16.688	16.688	84	0.0001

Note: df means degrees of freedom, SS means sum of squares deviations, MS means squares deviations, Est. Var means estimates of variance components, % means percentage of total variance contributed by each component, P-value means probability value.

**Table 10:** Analysis of genetic differentiation among accessions of *Lathyrus sativus* based on geographic origins by AMOVA.

Source of variation	df	SS	MS	Est. Var.	%	P-value
Geographic origins in Cultivars						
Among Pops	2	100.055	50.027	0.437	3%	0.0001
Within Pops	263	4087.547	15.542	15.542	97%	0.0001

Note: df means degrees of freedom, SS means sum of squares deviations, MS means squares deviations, Est. Var means estimates of variance components, % means percentage of total variance contributed by each component, P-value means probability value.

## Discussion

### Use of genetic diversity

Grasspea, as a neglected and underutilized species, is very popular among the resource poor farmers in marginal areas due to the ease with which it can be grown successfully under adverse agro-climatic conditions without much production inputs [5]. Genetic diversity is a source of traits for increased agricultural production and resistance to biotic and abiotic stresses [31]. Knowledge of genetic diversity will assist germplasm utilization in *Lathyrus sativus* breeding, and more climate-resilient varieties would be bred in the near future. There may be opportunities to exploit wiser genetic diversity in grasspea by combining germplasm between Asia and Africa/Europe, especially taking note of eco-geographical origins for complementation of extreme stress traits for drought tolerance, reproductive heat stress and salinity, for the breeding demands of specific target environments.

Further such exploration of diversity could include the more closely related *L*. *sativus* relatives which have more limited geographic range in cultivation, and attention to sources of low toxin to reduce the risk of poisoning in situations where grasspea is a major component of human diet.

### Comparison of grasspea genetic diversity

EST-SSRs have been used to detect the variability in grasspea accessions and to evaluate genetic diversity [11,12]. These markers were developed from *Lathyrus sativus* and transferable EST-SSRs from *L*. *japonicus* L. and *Medicago truncatula* respectively and the number was limited. In this study, the SSRs were developed by NGS sequencing of *L*. *sativus* genomic DNA [13]. Compared with the previous study [11,12], the genetic diversity of 283 accessions was higher, as the average allele number per locus was 8.6, and the average PIC value was 0.4817. In comparison with the *L*. *sativus* relatives, the cultivated germplasm, which came from Africa, Europe, Asia, and ICARDA, had much wider diversity than local germplasm, such as Ethiopia [12] and Italy [11], respectively. The level of polymorphism detected with genomic-SSRs was higher than that of EST-SSRs matching with the previous reports [32,33].

### Possibility of Genetic Flow

All the *Lathyrus* accessions were divided into three subgroups, under cultivated subgroups the accessions were classified according to geographical origins. The *Lathyrus sativus* relatives were separated from *L*. *sativus* clearly. Within the cultivated species, European and African accessions were aggregated together, and partially overlapped with some Asian accessions due to possibility of flow between the two subgroups. For example, Island Cyprus and ICARDA located in Asia, but 21 and 13 accessions were divided into European and African subgroup and only 1 and 2 accessions consisted to Asian subgroup, respectively [34,35].

### Richness of genetic diversity

The PCA of cultivated accessions by geographical distribution indicates that the first two principal components explained over 72% of the total genetic variation. Although most European and African materials flowed together, Asian accessions dispersed in much more extensive scope, as the PCA indicated (Fig. 6). More interestingly, the eight accessions from Bangladesh were relatively separated from others, as showed in Fig. 6. It means that the genetic diversity of cultivated accessions of grasspea originated from Asia is much richer than that from Europe and Africa.

### Genetic relationship and origin of *Lathyrus sativus*

Our accessions used in this study occupied vast territories of Southwestern, Western and Eastern Asia. It also occurred on isolated sites in Africa (Ethiopia and Eritrea). The European accessions were widespread throughout Southern and partly Central Europe, penetrating to the northern coast of Africa (Algeria, Morocco and Tunisia). The result of structure demonstrated that *Lathyrus sativus* divided into 2 population (Fig. 3). One contained 79 accessions and most of them distributed in Asia. The other included 187 accessions and most of them came from European and African countries. The UPGMA dendrogram (Fig. 4) also supported this hypothesis that there was a smaller genetic distance between African and European accessions than that of Asian accessions. AMOVA based on geographic origins (cultivated species divided into Asian, African, and European accessions) revealed that, in the total genetic variance, geographic-related variance was very limited (Table 10). Although Vavilov described Central Asia and Abyssinia as the centers of origin for *L*. *sativus* [36], our research results based on genotyping method partially supported the hypothesis that India together with adjacent areas was the primary centre of origin [37] which based on traditional phenotyping method. In conclusion, the natural distribution of *L*. *sativus* was obscured by cultivation, making it difficult to precisely locate its center of origin as described by Singh et al [38].
