# Correction: Genetic Diversity of Grasspea and Its Relative Species Revealed by SSR Markers

**DOI:** 10.1371/journal.pone.0126453

**Published:** 2015-04-17

**Authors:** The PLOS ONE Staff

Dr. John P. Hart is not included as the Academic Editor of this article. He should be listed as the Academic Editor and is affiliated with the New York State Museum, UNITED STATES.
